# GWAS for the composite traits of hematuria and albuminuria

**DOI:** 10.1038/s41598-023-45102-6

**Published:** 2023-10-23

**Authors:** Sarah A. Gagliano Taliun, Ian R. Dinsmore, Tooraj Mirshahi, Alexander R. Chang, Andrew D. Paterson, Moumita Barua

**Affiliations:** 1 Department of Medicine and Department of Neurosciences, Université de Montréal, Montréal, QC Canada; 2 Montréal Heart Institute, Montréal, QC Canada; 3 Department of Genomic Health, Geisinger, Danville, PA USA; 4 Department of Population Health Sciences, Center for Kidney Health Research, Geisinger, Danville, PA USA; 5 Department of Nephrology, Geisinger, Danville, PA USA; 6 Divisions of Epidemiology and Biostatistics, Dalla Lana School of Public Health, Toronto, ON Canada; 7 Genetics and Genome Biology, Research Institute at the Hospital for Sick Children, Toronto, ON Canada; 8 Institute of Medical Sciences, University of Toronto, Toronto, ON Canada; 9 Division of Nephrology, University Health Network, Toronto, ON Canada; 10 Department of Medicine, University of Toronto, Toronto, ON Canada; 11 Toronto General Hospital Research Institute, 8NU-855, 200 Elizabeth Street, Toronto, ON M5G2C4 Canada

**Keywords:** Genomics, Nephrology

## Abstract

Our GWAS of hematuria in the UK Biobank identified 6 loci, some of which overlap with loci for albuminuria suggesting pleiotropy. Since clinical syndromes are often defined by combinations of traits, generating a combined phenotype can improve power to detect loci influencing multiple characteristics. Thus the composite trait of hematuria and albuminuria was chosen to enrich for glomerular pathologies. Cases had both hematuria defined by ICD codes and albuminuria defined as uACR > 3 mg/mmol. Controls had neither an ICD code for hematuria nor an uACR > 3 mg/mmol. 2429 cases and 343,509 controls from the UK Biobank were included. eGFR was lower in cases compared to controls, with the exception of the comparison in females using CKD-EPI after age adjustment. Variants at 4 loci met genome-wide significance with the following nearest genes: *COL4A4*, *TRIM27*, *ETV1* and *CUBN*. *TRIM27* is part of the extended *MHC* locus. All loci with the exception of *ETV1* were replicated in the Geisinger MyCode cohort. The previous GWAS of hematuria reported *COL4A3*-*COL4A4* variants and HLA-B*0801 within *MHC*, which is in linkage disequilibrium with the *TRIM27* variant (D′ = 0.59). *TRIM27* is highly expressed in the tubules. Additional loci included a coding sequence variant in *CUBN* (p.Ala2914Val, MAF = 0.014 (A), *p* = 3.29E−8, OR = 2.09, 95% CI = 1.61–2.72). Overall, GWAS for the composite trait of hematuria and albuminuria identified 4 loci, 2 of which were not previously identified in a GWAS of hematuria.

## Introduction

Hematuria is a sign of kidney disease and can be assessed via dipsticks/microscopy and captured by ICD classification codes from electronic medical records. However, there has been less focus on this trait compared to albuminuria and eGFR. Nonetheless, reports show that hematuria is associated with a higher risk of kidney failure in the Israeli, Korean populations and CRIC cohort^1–3^.

A clinical decision point in hematuria comes from determining its origin which can be glomerular, tubular, interstitial or urinary tract^4,5^. This in turn informs diagnostic possibilities, prognoses and management. Hematuria can be identified as being more likely to be glomerular when there is accompanying albuminuria, hypertension and/or reduced kidney function (eGFR^5–7^). When hematuria and albuminuria co-occur, the diagnostic possibilities still remain long but IgA nephropathy has traditionally been taught as the commonest primary glomerulonephritides in the adult population^8^.

In previous genome wide association studies (GWAS) of kidney traits that included hematuria using the UK Biobank and deCODE, overlapping signals were identified namely on chromosome 2 where the collagen IV genes responsible for Alport syndrome reside^9–13^. The specific variants driving the association from these reports were different, however, owing to methodologic differences including differing minor allele frequency thresholds^13^. To address study limitations, we recently reported a GWAS with an exclusive focus on hematuria enriched for glomerular causes using phecode 593 in the white British subset of the UK Biobank^14^. Cases were found to have higher systolic blood pressure, higher urinary albumin:creatinine (uACR) and lower eGFR compared to controls, supporting the successful enrichment for glomerular pathologies^14^. Regarding methodology, we performed imputation using a reference dataset with higher resolution at associated loci on chromosome 2 and applied conditional regional analysis to identify independent variants driving the statistical association. As a result, we identified 3 independent variants in *COL4A4*-*COL4A3* but also a locus at *MHC*, both of which previous studies have implicated in Alport syndrome and IgA nephropathy, respectively^15–17^. Four other loci were also discovered and replicated.

Although most GWAS analyze one trait at a time, generating a combined phenotype can improve power to detect loci influencing multiple traits. Multivariate methods, such as multi-trait analysis of GWAS (MTAG), exist to combine summary-level genetic variant data. However, the latter can result in decreased power (for example, due to the need to use external datasets to estimate correlation among variants) and carries assumptions^18^. Though there are few multi-trait GWAS to date, a recent study evaluated coronary artery disease (CAD) and peripheral artery disease (PAD), which share similar risk factors, frequently co-occur and are genetically correlated^19^. Multi-trait GWAS was performed on PAD, CAD and different combinations of risk factors including BMI, smoking, type 2 diabetes and cholesterol using data from the Million Veteran Program, CARDIoGRAMplusC4D, GIANT, GLCG and the UK Biobank^19^. This analysis resulted in 25 novel pleiotropic loci, and co-localization with single-tissue eQTLs identified candidate causal genes at 14 of the signals. Thus, we take an analogous approach to hematuria and report the results of a GWAS for the combined phenotype of hematuria and albuminuria in the UK Biobank. Given access to individual-level genotypes and phenotypic data in the UK Biobank cohort, we create our combined phenotype using individual-level data to maximize statistical power.

## Materials and methods

### Study design

All experiments were performed in accordance with the Declaration of Helsinki. The study was approved by the Toronto General Hospital Research Ethics Board (21-5361.0) and by the Montreal Heart Institute Research Ethics Board (2022-3104). The UK Biobank is guided by the Ethics Advisory Committee to conduct research that abides by ethical principles involving human studies (Ethics (ukbiobank.ac.uk)). The UK Biobank has approval from the North West Multi-centre Research Ethics Committee as a Research Tissue Bank (RTB) approval. Researchers with approved access operate under the RTB approval and do not require separate ethical approval. For the replication cohort, Geisinger MyCode, all patients provided written informed consent. The Geisinger institutional review board approved the study.

GWAS was performed using UK Biobank data to identify variants associated with the combined traits of hematuria and albuminuria in white British subjects from the UK Biobank. The white British subgroup has been identified by the UK Biobank based on both self-report as ‘white British’ and similar genetic ancestry based on principal components analysis of genotypes. The first four principal components of genetic ancestry computed in the white British subset were derived in cases and controls, which were then included in the model as covariates to adjust for population stratification. Using the first four principal components has been shown to adequately control genomic inflation in the UK Biobank white British subset for binary traits using a linear mixed model to account for case–control imbalance^20^.

### UK Biobank setting, whole genome genotyping and centralized quality control

This research has been conducted using the UK Biobank Resource under Application numbers 48839 (M.B.) and 66222 (S.A.G.T). The UK Biobank is a prospective cohort involving approximately 500,000 UK adults between the ages of 40–69 years at the time of recruitment, in whom genetic and phenotypic data are collected^21^. The UK Biobank performed array genotyping at the Affymetrix Research Services Laboratory. We used the UK Biobank centrally imputed data^21^ that used the Haplotype Reference Consortium (HRC) data, consisting of primarily European genetic ancestry individuals, along with the merged UK10K and the 1000 Genomes project phase 3 reference panels^22,23^. When a genetic variant was present in both panels, the HRC imputation was used. The UK Biobank’s quality control procedure for samples and variants (either on the genotype array or imputed) have been described in detail^21^. In brief, for array variants, Affymetrix applied a custom genotype calling pipeline and quality control filtering, and the UK Biobank applied further standard filtering including testing for batch effects, plate effects, departures from Hardy–Weinberg equilibrium, sex effects, array effects, and discordance across control replicates. A variant that failed at least one quality control metric was assigned missing genotype calls. Pre-filtering of variants was conducted before phasing and imputation. Additionally, the UK Biobank provides the imputation R^2^ (referred to as information measure in the UK Biobank documentation) and the minor allele frequency for post-GWAS filtering. The UK Biobank notes that an information measure of 0.3 in around 150,000 samples corresponds to an effective sample size of around 45,000. We present R^2^ and minor allele frequency for all significant results.

### TOPMed-imputation of chromosome 2:226000000-228000000 region (GRCh38) of the UK Biobank

At the time of analysis, the TOPMed-imputed UK Biobank data was not yet available through the UK Biobank Research Analysis Platform. We thus used the Trans-Omics for Precision Medicine Program (TOPMed) imputation server, version r2 (https://imputation.biodatacatalyst.nhlbi.nih.gov/#!) to impute genetic variants in the chr2:226000000-228000000 region from the UK Biobank array data in batches of 20,000 individuals at a time^24^. The batches were subsequently merged. The panel includes 97,256 reference samples of diverse genetic ancestries and 308,107,085 genetic variants (distributed throughout the autosomes and chromosome X). Previous work has shown that imputing the UK Biobank to the TOPMed panel can help identify rare variants that are not present on other commonly-used reference panels in the white British subset^24^.

### Imputed *HLA*-haplotypes

The *HLA* region on chromosome 6 was further examined using the UK Biobank-provided *HLA*-imputed haplotypes (field 22,182; https://biobank.ctsu.ox.ac.uk/crystal/refer.cgi?id=182)^21^. The number of individuals included in the HLA reference panel varies depending on the HLA allele but ranges from 808 to 9120 (https://biobank.ctsu.ox.ac.uk/crystal/refer.cgi?id=182). Imputation was conducted using HLA*IMP:02 with modified settings using a set of genetically diverse reference datasets^25^. The imputation procedure and quality control steps have been described^26^. In brief, for each locus, only the reference individuals which had lab-based *HLA* types for that locus, and only the SNPs that were polymorphic and were typed in at least 98% of that set of individuals were included.

### Phenotype definition for case–control study

The R package PheWAS (https://github.com/PheWAS/PheWAS/blob/master/inst/doc/PheWAS-package.pdf) was used to map ICD9 and ICD10 codes (https://phewascatalog.org/phecodes_icd10cm) to phecode 593 “hematuria”^27^.

Composite cases had the following ICD9 or ICD10 codes: “hematuria”: 599.7 (ICD9), R31 (ICD10), “hematuria unspecified”: 599.70 (ICD9), R31.9 (ICD10) and “gross hematuria”: 599.71 (ICD9), R31.0 (ICD10) as well as an uACR ratio > 3 mg/mmol. Exclusion codes (i.e. an individual with any of the following codes were not included as either cases or controls) were: any genitourinary phecodes (individuals who have a phecode with the range 590–593.99). ICD9 and ICD10 codes underlying each phecode in this range were extracted to get the full list.

Controls did not have an ICD 9/10 code for hematuria and they had an uACR ≤ 3 mg/mmol.

uACR values were computed as (albumin in mg/L)/(creatinine in μmol/L/1000) from urine samples collected at baseline, where the numerator is UK Biobank field 30,500 (“microalbumin in urine”)^21^. Individuals marked as having urinary albumin < 6.7 mg/L (field 30,505) were assigned as having an urinary albumin value of 6.7 mg/L (Randox Bioscience, UK using immunoturbidimetric method and an analytical range from 6.7 to 200 mg/L (https://biobank.ndph.ox.ac.uk/showcase/showcase/docs/urine_assay.pdf)). The assay manufacturer for urine creatinine was Beckman Coulter, UK with enzymatic method and an analytical range from 88–44,200 µmol/L (https://biobank.ndph.ox.ac.uk/showcase/showcase/docs/urine_assay.pdf). Estimated glomerular filtration rate (eGFR) was calculated in two ways, using the CKD-EPI equation or as 100/cystatin C (mg/L) (https://biobank.ndph.ox.ac.uk/showcase/ukb/docs/serum_biochemistry.pdf)^28,29^.

Individuals were categorized as taking an angiotensin-converting enzyme inhibitor (ACEi) or an angiotensin receptor blocker (ARB) if they had at least one code corresponding to an ACEi/ARB medication from field 20,003 (treatment/medication code from verbal interview at the assessment centre) at the baseline visit. The specific ACEi/ARB medications from field 20,003 used are listed in Supplementary Table 1.

### Statistical methods

For the descriptive tables, all regression models were conducted in R (version 4.0.5). For each trait of interest (e.g., eGFR, BMI, etc.) we created a linear regression model with the trait as the outcome and the composite trait as the predictor to obtain the non-age-adjusted regression estimates (beta, standard error and corresponding *p* value) of the trait of interest on composite case–control status. For each trait of interest, we also constructed the same regression model but this time with age as an additional predictor to obtain the age-adjusted estimates. The models were constructed in females and males separately. To quantify whether a trait of interest differs significantly between the sexes, for each trait we also reported the sex-trait interaction term from a logistic model with the composite phenotype as the outcome and the following predictors: the trait of interest, age, sex and the interaction term for the trait of interest and sex. Values for the traits of interest were derived from the baseline visit.

SAIGE (v. 0.44.5) was used to test variants for association with the composite phenotype in the white British subset of the UK Biobank, where the effect allele is the non-reference allele^20^. For the X-chromosome, analyses were performed for males and females separately. For the Y-chromosome, variants genotyped on the UK Biobank arrays were analysed in males. Mitochondrial genetic variants genotyped on the UK Biobank arrays were also tested. Analyses were repeated using the final (July 2022) release of the UK Biobank whole-exome sequencing dataset to replicate protein coding results^30^.

***Variables***

Sex, birth year, and the first four genetic ancestry principal components computed on the white British subset were the covariates used in the analyses, except for the sex-stratified analyses on chromosome X and for the analyses on chromosome Y, for which sex was not included as a covariate. Related individuals were included given SAIGE uses a genetic relationship matrix to properly account for kinship. QR transformation (composition of the covariate matrix) of the covariate matrix was performed by default on non-binary covariates, such as birth year and principal components, to solve the linear least squares problem^31^. LocusZoom plots were generated^32^.

### Conditional analysis at significant loci

Conditional analyses at each the top loci were performed using the HRC/UK10K-imputed UK Biobank on the most significant variant at the locus using the condition flag within SAIGE.

### Phenotypic and genetic correlations between hematuria, continuous uACR, the composite trait and other related traits

We used genome-wide association summary statistics to estimate pairwise global genetic correlation between our in-house composite trait, ICD-based hematuria (phecode 593), continuous uACR, publicly available creatinine-based eGFR, and two impedance measurements (whole body fat mass and whole body fat-free mass) using LD score regression based on common HapMap autosomal SNPs and phase 3 of the 1000 Genomes Project European super-population as the linkage disequilibrium reference^33^. The summary statistics used for eGFR were the European-ancestry summary statistics from the meta-analysis conducted by Stanzick et al. 2021 which included European-ancestry individuals from the UK Biobank^34^. Other than the summary statistics for eGFR, all other summary statistics were derived exclusively from UK Biobank participants. The fat mass (field: 23,100) and fat-free mass (field: 23,101) summary statistics (measured from impedance) are made publicly available from the Neale lab from their round 2 single-variant association analyses testing the inverse rank-normal transformed trait in the white British UK Biobank subset. Phenotypic correlations were estimated by computing the Pearson correlation for the trait values in the UK Biobank white British subset.

### Replication of top association signals in the Geisinger MyCode DiscovEHR cohort

To replicate findings in the UK Biobank analysis, we used the European genetic ancestry subset of the Geisinger MyCode 175 k freeze cohort with exome sequencing and TOPMed-imputed genotypes from array data as an independent replication cohort. Geisinger MyCode is a health system-based cohort comprised of patients receiving care from Geisinger Health, encompassing central and northeast Pennsylvania, USA^35^. 149,492 participants of European genetic ancestry have whole-exome sequencing data and 147,813 also have TOPMed-imputed genotypes.

A sparse genetic relationship matrix (GRM) was created for the European-only subset using an LD-pruned subset of genetic variants in accordance with SAIGE recommendations (639,020 variants in total). Step 1 of SAIGE was performed using the sparse GRM with the same 639,020 variants. Cases and controls were defined in a prior MyCode paper on *COL4A3*^36^. In brief, cases had both dipstick hematuria (trace or greater on > 50% of urinalyses) and albuminuria (ACR 30 + mg/g [3.39 mg/mmol], protein/creatinine ratio 150 + mg/g [16.95 mg/mmol]; or if neither of the quantitative values were available: dipstick protein 1 + or greater on at least 2 urinalayses. Controls had neither dipstick hematuria nor albuminuria.

Step 2 of SAIGE (i.e. the association testing step) was then performed on both the whole-exome sequencing data and the TOPMed-imputated genotypes. For the TOPMed imputation, the following filters were used: MAF > 0.0001, INFO > 0.5, and AMPC >  = 0.95 (45,287,234 variants). Current age, sex, and the first ten principal components of genetic ancestry were included as covariates.

### Significance statement

In this study, we evaluated genetic loci associated with the composite traits of hematuria and albuminuria. In 2429 cases from the UK Biobank, 4 loci met genome-wide significance with the nearest genes: *COL4A4* causal for Alport syndrome, *TRIM27* which is part of the extended *MHC* locus, *CUBN* previously reported to be associated with albuminuria, and a novel locus in the intron of *ETV1*. Three loci excluding *ETV1* were replicated in the Geisinger MyCode cohort. Though *TRIM27* is highly expressed in the tubules, the identified variant is in linkage disequilibrium with a hematuria association we previously reported, HLA-B*0801. Clinically, our study guides clinicians as to possible etiologies for the combined trait of hematuria with albuminuria.

## Results

### Participants

In the UK Biobank, we identified 2429 (1112 females, 1,317 males) composite cases and 343,509 (181,031 females, 162,478 males) controls. The descriptive characteristics of the study population are presented in Table 1 and adjusted for age are presented in Supplementary Table 2. In the white British subset used for analysis, the overall rate for the composite trait of hematuria and albuminuria was 0.61% (1,112/182,031) and 0.80% (1317/163,478) for females and males, respectively. There were significantly more male than female cases (chi-square test *p* < 1E−5). Within the analysis subset with information on hematuria and albuminuria, the rate of glomerular hematuria was 4% (16,866/408,286) and for albuminuria (defined as an uACR ratio > 3 mg/mmol) was 11% (39,146/357,493) (Supplementary Table 3). Both female and male cases were significantly older than controls at baseline (T-test *p* value for both the female and male comparisons < 2.2E−16). Before and after adjustment for age, systolic and diastolic blood pressures were statistically significantly higher in cases than controls in both sexes. eGFR measured by CKD-EPI and cystatin C was lower in cases compared to controls, with the exception of the comparison in females using CKD-EPI after age adjustment. As expected given our case definition, uACR was significantly higher in cases compared to controls in both sexes. Cases and controls showed evidence of overlapping distributions in terms of principal component-space (Supplementary Fig. 1).

**Table 1:** Regression estimates for traits in the UK Biobank white British individuals used in the hematuria-uACR case–control analyses stratified by sex (UK Biobank field 22,001).

	Females		Males		Sex:trait interaction in case/control ~ trait + age + sex + trait:sex	
	Estimate (std error)	*P* value	Estimate (std error)	*P* value	Estimate	*P* value
Pulse rate, automated reading in bpm	1.1 (0.34)	2.1E−3	3.4 (0.35)	< 2.2E−16	1.4E−4 (2.5E−5)	2.4E−8
Systolic blood pressure, automated reading in mmHg	9.1 (0.62)	< 2.2E−16	8.5 (0.52)	< 2.2E−16	7.7E−5 (1.5E−5)	8.4E−7
Diastolic blood pressure, automated reading in mmHg	2.8 (0.32)	< 2.2E−16	2.0 (0.30)	5.2E−12	1.9E−5 (2.8E−5)	0.50
Standing height in cm	− 1.1 (0.19)	1.4E−8	− 1.1 (0.19)	1.8E−9	− 4.3E−5 (4.4E−5)	0.32
Weight in kg	− 0.68 (0.41)	0.1	3.7 (0.39)	< 2.2E−16	1.9E−4 (2.0E−5)	< 2.2E−16
BMI kg/m^2^	7.7E−2	0.62	1.5 (0.11)	< 2.2E−16	7.4E−4 (6.3E−5)	< 2.2E−16
Urinary Albumin-Creatinine Ratio in mg/mmol)*	9.5 (0.06)	< 2.2E−16	16.8 (0.11)	< 2.2E−16	− 3.7E−3 (9.5E−5)	< 2.2E−16
eGFR in mL/min/1.72m^2^**	− 1.9 (0.41)	1.76E−6	− 8.4 (0.36)	< 2.2E−16	− 3.4E−4 (2.3E−5)	< 2.2E−16
eGFR computed as 100/serum cystatin C (mg/L)	− 5.0 (0.55)	< 2.2E−16	− 12.9 (0.43)	< 2.2E−16	− 3.5E−4 (1.8E−5)	< 2.2E−16
Whole body fat mass (kg)	− 0.34 (0.30)	0.69	2.9 (0.23)	< 2.2E−16	3.8E−4 (3.2E−5)	< 2.2E−16
Whole body fat-free mass (kg)	− 0.67 (0.15)	9.9E−6	1.51 (0.21)	5.6E−13	2.3E−4 (4.8E−5)	1.9E−6

Sex-trait interaction for case/control status are also shown. Case/control counts and results from models with age-adjustment are in Supplementary Table 2.

*Individuals marked as having an urinary albumin value below 6.7 mg/L (field 30,505: “microalbumin in urine result flag”) were assigned as having an urinary albumin value of 6.7 mg/L. Then the ratio was computed as: (albumin in mg/L)/(creatinine in µmol/L/1000)).

**Estimated glomerular filtration rate (eGFR) calculation using the CKD-EPI equation or as 100/serum cystatin C (mg/L).

Male cases had both significantly higher BMI and whole body fat mass compared to male controls, after adjustment for age (Supplementary Table 2). However, there were no significant difference in these measures between female cases and controls. There was significant evidence for heterogeneity of these associations by sex.

### GWAS results

**Figure 1:**
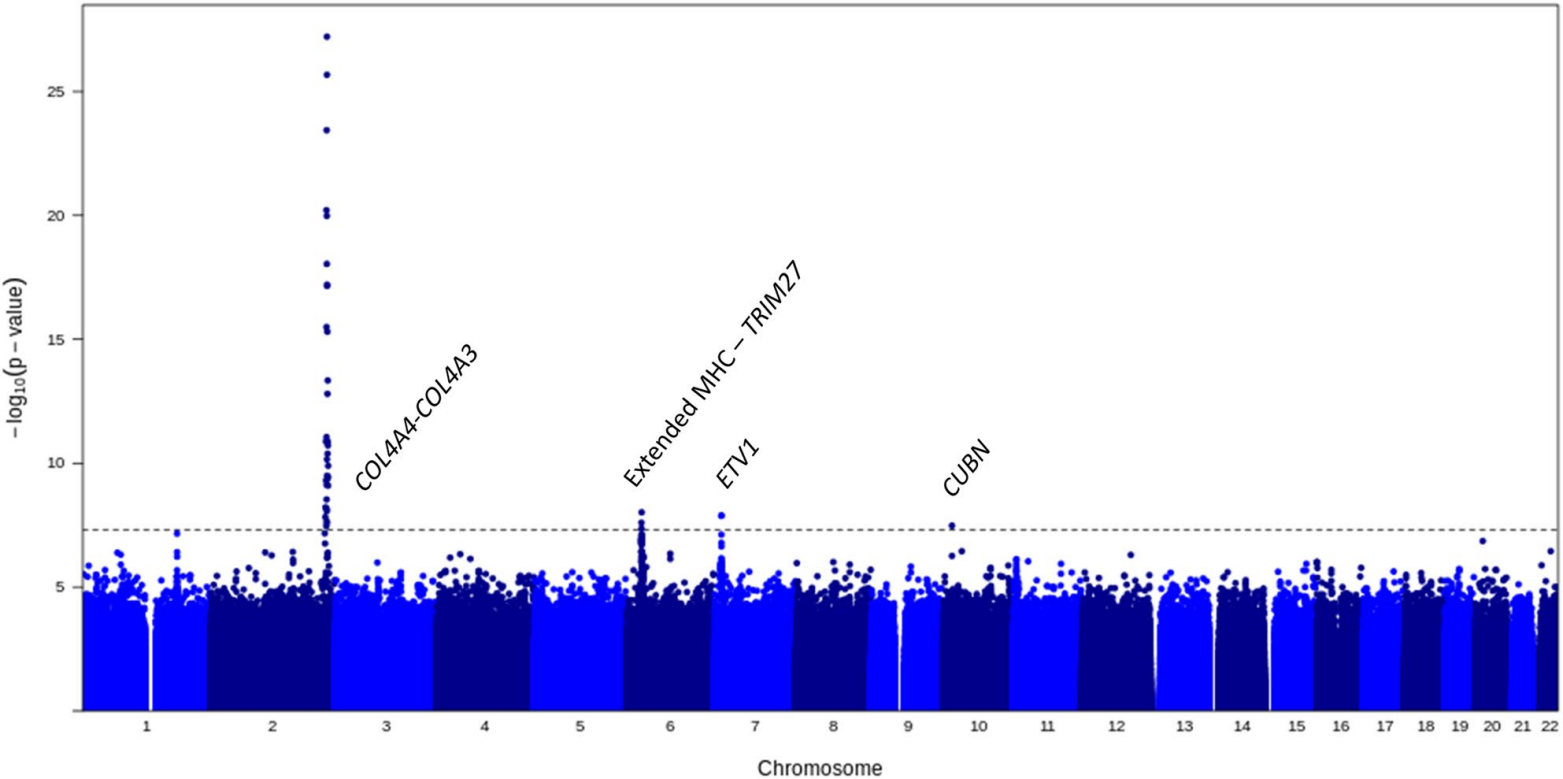
Manhattan Plot of Associated Signals for the composite outcome of Hematuria and Albuminuria using centrally HRC/UK10K-imputed UK Biobank Data created using R v. 4.0.5. Four statistically significant loci were identified. X-axis is the chromosome location from chromosome 1–22 and Y-axis is the − log_10_(*p* value). The horizontal grey hatched line represents the threshold for statistical significance at *p* = 5E−8.

We tested a total of 50,178,126 variants after imputation with HRC and UK10K with a minor allele count (MAC) > 20 located on either the autosomes or chromosome X for association with our composite trait. On the Y chromosome, 273 variants with a MAC > 20 on the UK Biobank genotyping array were tested for association. For mitochondrial variants, 239 on the array that passed the frequency threshold were tested for association for both sexes. There was no evidence for test statistics inflation (Supplementary Table 4, Supplementary Fig. 2). GWAS identified 4 loci (*p* < 5E−8, Fig. 1, Supplementary Fig. 3, Table 2, Supplementary Table 5). The following genes were closest to the strongest associated variants in each locus: *IRS1*/*COL4A4*, *TRIM27* within MHC, *ETV1* and *CUBN.* There were no genome-wide significant associations identified on chromosomes X or Y, the mitochondrial genome or with the UK Biobank imputed *HLA* haplotypes. All loci with the exception of the variant closest to *ETV1* replicated in the Geisinger MyCode cohort (Table 2, Supplementary Table 2). The case/control count was 8066/71,812, of which there were 5026 and 43,470 female cases and controls, respectively. The mean age of individuals was 59.7 ± 18.1 years. For the two coding variants (in *COL4A4* and *CUBN*), whole-exome sequencing association results were consistent with the TOPMed-imputed results.

**Table 2:** Top signals for composite trait GWAS (HRC-imputed data) and validation for protein-coding signals using whole exome sequencing (WES) data with replication in Geisinger MyCode cohort.

UK Biobank								Geisinger MyCode cohort					
Chr:pos:ref:alt_b37 (rsID)	Nearest gene	MAF (allele)	Imputation R^2^ or WES	Effect allele	Beta ± SE	OR (95% CI)	*P* value	MAF (allele)	Imputation R^2^ or WES	Effect allele	Beta ± SE	OR (95% CI)	*P* value
2:227917083_G/C (rs35138315)	COL4A4*	2.4E−4 (C)	0.57	C	3.21 ± 0.23	24.5 (15.8–38.8)	8.88E−31	6.3E−05 (C)	0.58	C	2.65 ± 0.52	14.21 (5.17–39.08)	1.35E−07
		4.2E−4 (C)	WES	C	3.21 ± 0.25	24.5 (15.1–40.4)	1.1E−38	8.9E−05 (C)	WES	C	2.59 ± 0.44	13.33 (5.63–31.58)	2.26E−09
6:28790373_C/CT (rs146924495)	*TRIM27*	0.18 (CT)	0.97	CT	− 0.22 ± 0.04	0.80 (0.74–0.87)	9.64E−09	0.15 (A)	1	A	− 0.11 ± 0.026	0.89 (0.85–0.94)	6.22E−06
7:13349195_G/A (rs146676616)	*ETV1*	0.009 (A)	0.97	A	0.96 ± 0.17	2.61 (1.87–3.63)	1.28E−08	0.013 (A)	0.9746	A	0.089 ± 0.076	1.09 (0.94–1.27)	0.242
10:16932384_G/A (rs45551835)	*CUBN*	0.014 (A)	1	A	0.74 ± 0.13	2.09 (1.61–2.72)	3.29E−08	0.013 (A)	0.997	A	0.42 ± 0.067	1.52 (1.33–1.73)	1.90E−10
		0.014 (A)	WES	A	0.58 ± 0.10	1.79 (1.47–2.17)	2.4E−08	0.013 (A)	WES	A	0.42 ± 0.067	1.52 (1.33–1.73)	1.90E−10

*WES* Whole-exome sequencing.

*HRC-imputed UK Biobank does not include the *COL4A4* Ser969Ter rare loss of function variant that is the top signal in the hematuria analysis (assessed in TOPMed-imputed data). Effect size estimates for the chromosome 2 variant are presented from the Firth test based on an unrelated subset of 1,983 cases and 281,636 controls as SAIGE produced unstable effect size estimates given the low allele frequency. MAF = minor allele frequency. For allele counts separately by case–control status, see Supplementary Table 5.

**A proxy (r^2^ > 0.9) was used in the replication cohort: 6:28824700_G/A (rs209181).

The strongest association was observed for rs71431010 on chromosome 2 (MAF = 0.00059 (A) in cases and MAF = 0.00037 in controls, *p* = 5.97E−28, OR = 22.6, 95% CI = 13.9–36.9), which is intergenic and the closest protein-coding gene is *IRS1*. We further investigated this region using a denser set of imputed genetic variants using the TOPMed imputation reference panel. The most significant variant in this region was *COL4A4* p.Ser969Ter; rs35138315 (chr2:227052367:G:C on build GRCh38; *p* = 8.88E−31; MAF = 0.0039 (C) in cases and MAF = 0.00022 in controls), which is the same top variant that we previously identified in our hematuria GWAS^14^. No variants in this region remained genome-wide significant after conditioning on the *COL4A4* p.Ser969Ter variant. Effect size estimates for the chromosome 2 variant in Table 2 are presented from the Firth test based on an unrelated subset of 1,983 cases and 281,636 controls as SAIGE produced unstable effect size estimates given the low allele frequency.

We validated association results for the two protein coding signals (*COL4A4* p.Ser969Ter and *CUBN* p.Ala2914Glu) using the whole-exome sequencing data from the UK Biobank (Table 2).

The second most significantly associated signal was an indel on chromosome 6 (*TRIM27*, rs146924495, MAF = 0.18 (CT), *p* = 9.64E−9, OR = 0.80, 95% CI = 0.74–0.87) within the extended MHC locus, where both *HLA* and non-*HLA* genes exist. This variant is in linkage disequilibrium (LD) with the HLA-B*0801 locus reported in the previous hematuria GWAS. Interestingly, *TRIM27* displays expression in the proximal tubule, distal convoluted tubule and collecting duct (staining in Supplementary Fig. 4, gene expression in Supplementary Table 6).

Association was also found with a missense variant in *CUBN* on chromosome 10 (rs45551835, G > A in genome sequence, p.Ala2914Val ((NP_001072.2) MAF = 0.014 (A), *p* = 3.29E−8, OR = 2.09, 95% CI = 1.61–2.72). The top variant at the *CUBN* locus in our composite GWAS (rs45551835) is one of the *CUBN* variants identified by Casanova et al. as being associated with uACR and it is in linkage disequilibrium (LD) (D′ = 1) with the other two *CUBN* variants reported by the group (rs141640975, rs45619139) in the UK Biobank based on high-coverage LD information from the European super-population of Phase 3 of the 1000 Genomes Project as implemented in the LDpair tool of the LDlink suite of web-based applications (Table 3)^37^.

**Table 3:** Linkage disequilibrium of rs45551835 with *CUBN* variants in Casanova et al. 2019 based on 1000 Genomes Project phase 3 European^37^.

Variant pair (minor allele, frequency)	R^2^	D′
rs45551835 (A, 0.01) and rs141640975 (A, 0.004)	0.0	1.0
rs45551835 (A, 0.01) and rs45619139 (G, 0.0905)	0.101	1.0

A novel association was also identified on chromosome 7 (rs146676616, MAF = 0.009 (A), *p* = 1.28E8, OR = 2.61, 95% CI = 1.87–3.63, nearest gene *ETV1*) (Table 2). However, this variant was not replicated in the Geisinger MyCode cohort.

No variants remain significant after conditioning on the most significant variant for the four genome-wide significant loci on chromosomes 2, 6, 7 and 10. Tissue/cell-specific expression at each locus is shown in Supplementary Table 6 and PheWAS results (indicating traits for which the variant-trait association *p* value is < 1E−3) are shown in Supplementary Table 7. The novel locus in an intron of *ETV1*, which has overall low expression in the kidney, appears to have the highest expression in the loop of Henle based on a single cell sequencing atlas (https://humphreyslab.com/SingleCell/displaycharts.php), the Human Protein Atlas and GTex v8 (Supplementary Table 6).

Comparison of effect sizes and *p* values for hematuria and/or uACR for *COL4A4*, *TRIM27*, *ETV1* and *CUBN* top variants are shown in Table 4 to assess the contribution of each trait to the composite phenotype. Based on the odds ratios, there is a stronger association of the *TRIM27* variant with the composite than either trait independently. The *COL4A4* variant is significantly associated with both traits (*p* < 7.95E−35 for hematuria and *p* < 4.0E−77 for uACR as a quantitative measure) and the composite (*p* < 8.88E−31). The *ETV1* intronic variant is significantly associated with just the composite (*p* < 1.28E−8) and has not been previously reported. The *CUBN* variant is predominantly associated with uACR (*p* < 1.22E−99). As another approach to dissect the associations for the two traits (hematuria and uACR), for each of the four top variants, we ran a logistic regression model in an unrelated UK Biobank analysis subset with the variant as the outcome and hematuria and uACR jointly as predictors as well as covariates sex, birth year and the first four principal components (Supplementary Table 8). For the *CUBN* variant, both hematuria and uACR were significant in the model (*p* < 2.5E−6 and *p* < 6.61E−4, respectively). For the p.Ser969Ter *COL4A4* variant, hematuria was significant (*p* < 2E−16), and uACR was not significant (*p* = 0.067). For the last two models (*TRIM27* and *ETV1* variants), hematuria was significant (*p* < 3.4E−6), but not albuminuria (*p* > 0.1).

**Table 4:** Comparison of effect sizes for the alternate allele and *p* values in hematuria and uACR GWAS for *COL4A4*, *TRIM27*, *ETV1* and *CUBN* top variants from the composite GWAS in the UK Biobank.

Top variant (nearest gene)	OR (95% CI); *p* value			
	Hematuria	uACR (inverse normal transformation of mg/mmol, continuous)	uACR (binary; case > 3 mg/mmol)	Composite
rs35138315 (*COL4A4)*	87.3 (47.9–159); *p* = 7.95E−35	4.83 (4.09–5.70); *p* = 4.0E−77	233.1 (116.7–465.7); *p* = 8.9E−54	24.5 (15.8–38.8); *p* = 8.88E−31
rs146924495 (*TRIM27*)	0.88 (0.85–0.90); *p* = 3.6E−17*	0.99 (0.99–1.00); *p* = 0.20	0.98 (0.96–0.99); *p* = 0.015	0.80 (0.74–0.87); *p* = 9.64E−09
rs146676616 (*ETV1*)	1.12 (0.99–1.26); *p* = 0.068	1.02 (0.99–1.04); *p* = 0.12	1.02 (0.99–1.11); *p* = 0.56	2.61 (1.87–3.63); *p* = 1.28E−08
rs45551835 (*CUBN*)	1.12 (1.02–1.23); *p* = 0.024	1.21 (1.19–1.23); *p* = 1.22E−99	1.56 (1.45–1.66); *p* = 2.49E−39	2.09 (0.74–0.87); *p* = 3.29E−08

*Association statistics for rs209181 which is in high linkage disequilibrium (r^2^ > 0.8) with the top variant, indel rs146924495, are shown for hematuria.

### Phenotypic and genetic correlations between hematuria, continuous uACR, the composite phenotype and other related traits

We examined the phenotypic correlation of these 3 traits to one another as well as their correlation with other traits using participants from the white British UK Biobank (Supplementary Table 9). Expected phenotypic correlations between hematuria, continuous uACR, eGFR and the composite traits were present. Additionally, whole body fat mass and whole body fat free mass were each phenotypically correlated with hematuria, continuous uACR and the composite traits. Fat mass and fat free mass were positively correlated with hematuria. Fat mass was positively and fat free mass was negatively correlated with continuous uACR. The composite trait was positively correlated with fat mass and fat free mass.

Assessing global genetic correlation between pairs of traits can be helpful to inform on potentially shared genetic underpinnings. Global genetic correlations between pairs of traits were estimated from summary statistics (Supplementary Table 9). Hematuria was not significantly genetically correlated with uACR or eGFR. There was a significant positive genetic correlation between continuous uACR and eGFR. There was negative genetic correlation between both fat mass and fat free mass with uACR and positive genetic correlation with whole body fat mass and hematuria. Fat mass was positively genetically correlated with the composite trait, but fat free mass was not significantly genetically correlated with the composite trait.

## Discussion

Here we present a dedicated GWAS for the composite traits of hematuria and albuminuria in the white British subset of the UK Biobank. We identified four loci as genome-wide significantly associated. For the loci closest to *COL4A4* and *CUBN*, these have been previously reported in GWAS of hematuria (*COL4A4*) and albuminuria (*COL4A4, CUBN*)^38–41^. The association with nearest gene *TRIM27* is within the MHC locus, which has been implicated in IgA nephropathy and steroid sensitive nephrotic syndrome studies^42–46^. The top *TRIM27* variant is in LD with HLA-B*0801 that was reported in our previous hematuria GWAS using the same dataset^14^. We identified one novel association in an intron of *ETV1*, but *ETV1* has low expression in the kidney, and was not replicated in the Geisinger MyCode cohort.

The UK Biobank is linked to electronic health records (EHR) to facilitate GWAS using International Classification of Diseases (ICD) codes. Alternatively, phecodes are a high-throughput tool that aggregates ICD codes and are designed to rapidly define phenotypes using EHR data that can also be used for GWAS. Previous studies have been performed to confirm the precision of phecodes to define phenotypes, though not for hematuria^47,48^. In this study, we took advantage of phecodes to define hematuria that is enriched for glomerular causes that has occurred over the time period during which ICD codes have been used in UK National Health Service databases, which is typically over decades. Alternatively, hematuria can be defined by urine dipsticks and/or microscopy which was not available in the UK Biobank. In our previous work, we demonstrated that the majority of genetic associations identified for hematuria in the UK Biobank were confirmed in GWAS using urinalysis data from deCODE^9,14^. Our current work with the Geisinger MyCode cohort also confirms this approach^14^. We note that there is considerable range in the hematuria rates among cohorts: 4% in the UK Biobank (phecode-based), 22% in Geisinger (dipstick-based), and 49% (mild/moderate/severe) in deCODE (dipstick-based). The variability is likely in at least part due to the different sensitivity and specificity of each phenotyping approach.

By contrast, the uACR comes from a single cross-sectional measure obtained at UK Biobank baseline examination. Combining phenotypes offers multiple advantages including phenotype precision, efficiency in detecting genetic variants missed by univariate screening and identifying pleiotropic effects for associated loci^49^.

Our results confirm phenotypic precision, identifying one locus well known to have pleiotropic causality for hematuria and albuminuria which is *COL4A4*, implicated in a monogenic disorder called Alport syndrome, reported to follow autosomal dominant and recessive inheritance patterns^13,50–54^. For example, rs35138315 (*COL4A4* p.Ser969Ter) has been reported in GWAS of albuminuria (UK Biobank), GWAS of hematuria (UK Biobank) and in typical forms of autosomal recessive Alport syndrome^13^. Furthermore, we identify three additional loci/variants that were not discovered in our previous GWAS of the single trait hematuria in the UK Biobank. These include variants in or closest to *TRIM27*, *CUBN* and *ETV1*, though the latter was not replicated. Possible reasons for the lack of replication of the *ETV1* variant could be lack of power and/or different phenotyping approaches. The variant closest to *TRIM27* is intergenic and is in LD with an association with HLA-B*0801, that we previously reported in a hematuria GWAS in the white British subset of the UK Biobank. TRIM27 itself is expressed along the tubular segments (staining in Supplementary Fig. 4 and gene expression in Supplementary Table 6), but the mechanisms by which this gene would cause hematuria is unclear given its localization. Of note, however, this *TRIM27* indel was not part of the analysis in the hematuria GWAS which only included biallelic SNPs.

For *CUBN*, the findings potentially expand the phenotypic spectrum which has been previously reported to include albuminuria and has a modest association with hematuria with an effect size of 1.12 (1.02–1.23; *p* = 0.024; Table 4) and was replicated in the Geisinger MyCode cohort (Table 2)^10,37–39^. Prior studies suggest that the endocytic receptors megalin and cubulin, located in the proximal tubule, is responsible for renal uptake of hemoglobin and myoglobin^55^. Myoglobinuria is a consequence of muscle injury, called rhabdomyolysis, which can cause acute kidney injury. Myoglobin reacts positively for blood by urine dipstick and so it is possible that the association is with myoglobinuria and not hematuria. Our analysis also shows that this missense variant in *CUBN* is in linkage disequilibrium with other intronic *CUBN* variants reported in GWAS of uACR based on imputation using the 1000 Genomes project (Table 3). For this *CUBN* missense variant rs45551835, it may have been missed by previous studies due to its low frequency. LD data demonstrates that two *CUBN* variants previously reported by Casanova et al. are on the same haplotype as rs45551835^37^. D′ is more informative than r^2^ in this context given the differing allele frequencies between the variants. Allele specific expression on this rare variant is not available in GTEx.

We also noted differences in clinical characteristics between cases and controls. Both female and male cases were older than their respective controls (Table 1). Blood pressure was higher and eGFR was lower in cases compared to controls by sex both before and after age adjustment, with the exception of the comparison in females using CKD-EPI after age adjustment (Table 1, Supplementary Table 2). Overall cases had mild renal disease with uACRs of 4.54 mg/mmol (3.58–7.78) in females and 6.13 mg/mmol (3.95–13.6) in males. These uACRs, however, are likely underestimated because there were approximately twice as many cases as controls taking ACEi and/or ARB (Supplementary Table 2). A second issue is of misclassification. Given that we used an uACR threshold to define cases, individuals on ACEi and/or ARB may fall below the threshold but without the medication may have met case criteria. After performing the analysis excluding individuals taking an ACEi and/or ARB, we obtained similar results though only 2 loci at chromosome 2 and 6 were statistically significant as a result the case number falling substantially (Supplementary Table 10). There are more male than female cases at this mild end of the spectrum, though more severe cases will also be captured (Table 1).

Though hematuria and albuminuria had a marginal genetic correlation (*p* = 0.005, Supplementary Table 9), this is a combined clinical trait seen routinely by nephrologists including in the rare disorders of Alport syndrome, IgA nephropathy, membranous nephropathy and proliferative glomerulonephritides^5–7^. Hematuria can be identified as being more likely to be glomerular when there is accompanying albuminuria, hypertension and/or reduced kidney function (eGFR), with known mechanisms driving this association^5–7^.

Additionally, in our previous hematuria GWAS, cases were found to have increased uACR compared to controls^14^. The lack of genetic correlation could be due to the limits of the tool, which only examines genome-wide global correlation based on common variants, which will miss correlations amongst rarer variants or regional rather than genome-wide correlations. With regard to possible local rather than global correlations, work by deCODE, for instance, identified a rare deletion–insertion spanning exons 16 and 17 of *COL4A3* associated with increased risk of hematuria and also an increased risk of proteinuria in their Icelandic dataset^9^. Further, both measures, uACR and hematuria have a margin of error. The uACR measurement is from a single untimed sample, and the hematuria phecode used to classify cases and controls is derived from inclusion and exclusion ICD patient discharge codes and thus has a complex relationship to measured hematuria. When there are measurement errors in traits and pleiotropy exists, then combinations of traits can improve power. Indeed, particular composites are routinely assessed in GWAS to improve power, including body mass index, phecodes and uACR^38,56,57^.

For the albuminuria trait, we selected a routine uACR threshold. However, different thresholds may be needed to identify signals at different loci depending on the extent to which these contribute to albuminuria, and they may also vary by sex. This challenge may be overcome by combining the binary traits of hematuria with continuous uACR.

Limitations of our study include analyzing only the white British subset given that this is the largest ancestral group but restricts generalizability. In the future, sequencing data (as opposed to genotyping/imputation) will provide greater resolution at associated loci, though LD may still affect the ability to identify the specific variants driving the statistical association. Using a composite trait decreases case count so replication of novel loci will have to be in cohorts that are adequately powered. In our previous GWAS for the single trait of hematuria, we used deCODE (n = 151,677) for replication but the cohort size is significantly smaller than the UK Biobank. In general, however, even with replication GWAS signals are associations and cannot be said to be causative without additional supportive evidence including tissue expression and importantly functional studies. Finally, we used the first four principal components of genetic ancestry which has been shown to adequately control genomic inflation in the UK Biobank white British subset for binary traits using a linear mixed model to account for case–control imbalance^20^. After inclusion of ten principal components for the white British subjects, all genome-wide significant loci (and top variants) remained the same, with just slightly different effect sizes and *p* values (comparisons shown in Supplementary Table 11).

We extend our previous hematuria GWAS by evaluating the genetic associations for the combined trait of hematuria and uACR in the white British subset of the UK Biobank and in the Geisinger MyCode cohort. Analyses identified a variant in *COL4A4* and within the extended MHC locus, the latter of which is in LD with a nearby signal in *HLA-B* from the hematuria GWAS. Additionally, a variant in *CUBN* is also identified and replicated. The power of analyzing the combined trait includes identifying pleiotropic effects and uncovering novel statistical associations.

### Supplementary Information

Supplementary Information.

Supplementary Table 2.

## Data Availability

The UK Biobank analysed during the current study are available to bona fide researchers via application directly to the UK Biobank (https://www.ukbiobank.ac.uk). All participants gave broad consent to use of their anonymised data and samples for any health-related research. The datasets generated and/or analysed during the current study are available. The association summary statistics from our composite HRC-imputed GWAS of hematuria and uACR including all single-variant results are available for download at https://my.locuszoom.org/gwas/736976/. Web Resources: Other web resources included: https://my.locuszoom.org, http://locuszoom.org/, https://biobank.ndph.ox.ac.uk/showcase/, https://github.com/weizhouUMICH/SAIGE, https://databases.lovd.nl/shared/genes/COL4A4, https://databases.lovd.nl/shared/genes/COL4A3, https://imputation.biodatacatalyst.nhlbi.nih.gov/#!, http://www.haplotype-reference-consortium.org/data-access, https://github.com/PheWAS/PheWAS/blob/master/inst/doc/PheWAS-package.pdf, https://phewascatalog.org/phecodes, https://phewascatalog.org/phecodes_icd10cm, https://ldlink.nci.nih.gov, http://www.nealelab.is/uk-biobank, https://pheweb.org/UKB-TOPMed/.
