# Author Correction: GWAS for the composite traits of hematuria and albuminuria

**DOI:** 10.1038/s41598-023-50577-4

**Published:** 2024-01-11

**Authors:** Sarah A. Gagliano Taliun, Ian R. Dinsmore, Tooraj Mirshahi, Alexander R. Chang, Andrew D. Paterson, Moumita Barua

**Affiliations:** 1 Department of Medicine and Department of Neurosciences, Université de Montréal, Montréal, QC Canada; 2 Montréal Heart Institute, Montréal, QC Canada; 3 Department of Genomic Health, Geisinger, Danville, PA USA; 4 Department of Population Health Sciences, Center for Kidney Health Research, Geisinger, Danville, PA USA; 5 Department of Nephrology, Geisinger, Danville, PA USA; 6 Divisions of Epidemiology and Biostatistics, Dalla Lana School of Public Health, Toronto, ON Canada; 7 Genetics and Genome Biology, Research Institute at the Hospital for Sick Children, Toronto, ON Canada; 8 Institute of Medical Sciences, University of Toronto, Toronto, ON Canada; 9 Division of Nephrology, University Health Network, Toronto, ON Canada; 10 Department of Medicine, University of Toronto, Toronto, ON Canada; 11 Toronto General Hospital Research Institute, 8NU-855, 200 Elizabeth Street, Toronto, ON M5G2C4 Canada

Correction to: *Scientific Reports* 10.1038/s41598-023-45102-6, published online 23 October 2023

The Supplementary Table 2 file published with this Article contained errors in the bottom half of the descriptive data for the Geisinger cohort where the outcome and predictor variables were incorrectly swapped. The original Supplementary Table 2 file is provided below.

These errors have now been corrected in the Supplementary Table 2 file that accompanies the original Article.

### Supplementary Information

Supplementary Table 2.

