# Supplementary material for: GWAS for the composite traits of hematuria and albuminuria
Source: Sci Rep. 2023 Oct 23;13:18084. doi: 10.1038/s41598-023-45102-6 (PMC10593773; doi:10.1038/s41598-023-45102-6)
Supplement: Supplementary file 1 — Supplementary Information. [file 41598_2023_45102_MOESM1_ESM.docx]

**Supplementary Table 1. ACEi and/or ARBs from field 20003 considered in primary analysis using UK Biobank in Supplementary Table 2.**

| **ACE inhibitor or ARB** |
| --- |
| acepril 12.5mg tablet |
| candesartan cilexetil |
| capoten 12.5mg tablet |
| captopril |
| captopril+hydrochlorothiazide 25mg/12.5mg tablet |
| cilazapril |
| co-diovan 80mg/12.5mg tablet |
| coversyl 2mg tablet |
| coversyl plus 4mg/1.25mg tablet |
| cozaar 25mg tablet |
| cozaar half strength 25mg tablet |
| cozaar-comp 50mg/12.5mg tablet |
| diovan 40mg capsule |
| enalapril |
| enalapril maleate+hydrochlorothiazide 20mg/12.5mg tablet |
| eprosartan |
| felodipine+ramipril |
| fosinopril |
| hypapril 12.5mg tablet |
| irbesartan |
| irbesartan+hydrochlorothiazide 150mg/12.5mg tablet |
| lisinopril |
| lisinopril+hydrochlorothiazide 10mg/12.5mg tablet |
| losartan |
| losartan potassium+hydrochlorothiazide 50mg/12.5mg tablet |
| micardis 20mg tablet |
| micardisplus 40mg/12.5mg tablet |
| moexipril |
| olmesartan |
| perindopril |
| perindopril+indapamide |
| quinalapril+hydrochlorothiazide 10mg/12.5mg tablet |
| quinapril |
| ramipril |
| telmisartan |
| telmisartan+hydrochlorothiazide 40mg/12.5mg tablet |
| tensopril 12.5mg tablet |
| teveten 300mg tablet |
| trandolapril |
| trandolapril+verapamil hydrochloride |
| valsartan |
| valsartan+hydrochlorothiazide 80mg/12.5mg tablet |
| valsartan+hydrochlorothiazide 80mg/12.5mg tablet |
| valsartan+hydrochlorothiazide 80mg/12.5mg tablet |
| zestril 2.5mg tablet |

**Supplementary Table 2 – Descriptive characteristics, adjusted for age; see Excel file**

**Supplementary Table 3.** Counts of white British UK Biobank individuals with either hematuria and/or uACR >3 mg/mmol in the UK Biobank

| **Description** | **Number of individuals** |
| --- | --- |
| Hematuria and uACR >3 mg/mmol (cases in GWAS) | 2,429 |
| Hematuria and no uACR >3 mg/mmol (excluded from GWAS) | 14,437 |
| uACR >3 mg/mmol and no Hematuria (excluded from GWAS) | 36,649 |
| No Hematuria and no uACR >3 mg/mmol (controls in GWAS) | 343,509 |

**Supplementary Table 4**. Genomic control (GC) lambda for allele frequency bins in the UK Biobank.

| **Allele frequency bin** | **GC lambda** |
| --- | --- |
| (0.05, 0.5] | 1.02 |
| (0.005, 0.05] | 1.02 |
| (0.001, 0.005] | 1.02 |
| [2.8E-5, 0.001] | 0.88 |

**Supplementary Table 5**. Effect allele frequency and counts in cases and controls at significantly associated loci in the UK Biobank.

| Nearest gene (variant) | Effect allele | Cases | | Controls | |
| --- | --- | --- | --- | --- | --- |
|  |  | Allele frequency (%) | Counts* | Allele frequency (%) | Counts* |
| *COL4A4 +* (rs35138315) | C | 0.39 | 19 | 0.022 | 148 |
| *TRIM27* (rs146924495) | CT | 15.24 | 740 | 18.39 | 126,102 |
| *ETV1* (rs146676616) | A | 1.77 | 86 | 0.91 | 6,246 |
| *CUBN* (rs45551835) | A | 2.37 | 116 | 1.38 | 9,466 |

*Counts are rounded as imputed dosages rather than hard genotypes were used.

+ HRC-imputed UK Biobank does not include the *COL4A4* Ser969Ter rare loss of function variant that is the top signal in the hematuria analysis (assessed in TOPMed-imputed data).

**Supplementary Table 6**. Cell and tissue specific expression of loci associated with haematuria + albuminuria GWAS. PT=proximal tubule, LH=loop of Henle, DCT=distal convoluted tubule, CNT=connecting tubule, PC=principal cell, IC-A=intercalated cell A, CD=collecting duct, EC=endothelial cell

| Nearest gene | snRNAseq in healthy human adult kidney (4,524 nuclei; humphreyslab.com) | The Human Protein Atlas single cell (proteinatlas.org)  nTPM shown in parentheses | The Human Protein Atlas normal kidney tissue staining | GTex V8  Median TPM in kidney |
| --- | --- | --- | --- | --- |
| *COL4A4* | Podocyte, PT, LH, DCT, CD:IC | PT (1.7-12.5), DCT (1.9), CD (46) | Not done | 8 (cortex), 7.26 (medulla) |
| *TRIM27* | CD:IC, CD:PC, PT, LH, podocytes | PT (21.5-34.4), DCT (21.5), CD (29.5) | Not detected in glomeruli, high expression in tubules | 16.77 (cortex), 27.29 (medulla) |
| *ETV1* | LH, CD:IC, podocytes, CD:PC, PT | PT (0.4-5.3), DCT (5.9), CD (3.7) | Not done | 0.860 (medulla) |
| *CUBN* | PT, CD:IC, podocytes, DT, LH | PT (154.6-723.3), DCT (13.7), CD (5.5) | Not detected in glomeruli, high expression in tubules | 10.3 (cortex), 2.92 (medulla) |

**Supplementary Table 7**: Association of 4 variants from hematuria + albuminuria GWAS with other renally relevant traits from the TOPMed-imputed UK Biobank dataset. Traits with p-value <1E-3 are shown. Source is https://pheweb.org/UKB-TOPMed/. Effect size is to alternate allele.

| Gene, SNP | Phenotype | P-value | Beta (SE) | Number of samples (cases/controls) |
| --- | --- | --- | --- | --- |
| *COL4A4* p.Ser969Ter rs35138315 | Hematuria | 8.8E-49 | 4.5 (0.31) | 16235/378356 |
|  | Essential hypertension | 6.8E-7 | 0.61 (0.12) | 77465/328796 |
|  | Hypertension | 8.0E-7 | 0.61 (0.12) | 77714/328796 |
|  | Other symptoms/disorders of the urinary system | 8.9E-5 | 3.6 (0.92) | 1387/396050 |
|  | Retinal detachment with retinal defect | 9.6E-5 | 3.6 (0.92) | 1387/396050 |
|  | Nephritis and nephropathy with pathological lesion | 1.3E-4 | 11 (2.9) | 252/395835 |
|  | Chronic glomerulonephritis, NOS | 1.4E-4 | 4.6 (1.2) | 877/395835 |
|  | Nephritis; nephrosis; renal sclerosis | 2.0E-4 | 3.2 (0.85) | 1531/395835 |
|  | Glomerulonephritis | 4.3E-4 | 3.7 (1.1) | 1052/395835 |
|  | Proteinuria | 5.6E-4 | 7.4 (2.1) | 337/406470 |
|  | Myopia | 7.5E-4 | 3.3 (0.97) | 1249/404788 |
|  | Chronic renal failure | 8.4E-4 | 2.0 (0.60) | 2649/395832 |
|  | Retinal detachments and defects | 9.4E-4 | 1.8 (0.55) | 3244/396051 |
|  |  |  |  |  |
| *TRIM27*  rs209181* | Hematuria | 1.3E-17 | -0.13 (0.015) | 16235/378356 |
|  | Disorders of iron metabolism | 2.0E-6 | -0.34 (0.071) | 665/405082 |
|  | Nephrotic syndrome without mention of glomerulonephritis | 9.9E-4 | 0.29 (0.087) | 467/395834 |
|  |  |  |  |  |
| *ETV1* rs146676616 | Optic neuritis/neuropathy | 1.3E-3 | 2.5 (0.77) | 125/399529 |
|  | Other disorders of soft tissues | 1.4E-3 | -0.31 (0.098) | 6024/377183 |
|  | Myeloid leukemia, acute | 3.7E-3 | 1.3 (0.44) | 328/402714 |
|  | Placenta previa and abruptio placenta | 4.8E-3 | 0.61 (0.22) | 1268/212249 |
|  | Flat foot | 5.0E-3 | 1.7 (0.59) | 189/393241 |
|  | Prostatitis | 5.2E-3 | 0.54 (0.19) | 1656/169005 |
|  | Disorders resulting from impaired renal function | 5.5E-3 | 2.4 (0.85) | 99/395833 |
|  | Other dyspnea | 6.1E-3 | 0.68 (0.25) | 912/398129 |
|  | Insomnia | 6.5E-3 | 1.7 (0.63) | 163/401998 |
|  | Diseases of the salivary glands | 7.2E-3 | 0.77 (0.28) | 693/401593 |
|  |  |  |  |  |
| *CUBN* rs45551835 | Malignant neoplasm of liver, primary | 1.5E-3 | 1.7 (0.52) | 158/391483 |
|  | Sarcoidosis | 2.0E-3 | 0.81 (0.26) | 539/400947 |
|  | Other symptoms of respiratory system | 3.4E-3 | -0.19 (0.064) | 9061/398129 |
|  | Displacement of intervertebral disc | 3.5E-3 | 0.78 (0.27) | 512/390232 |
|  | Hypertension | 4.2E-3 | 0.077 (0.027) | 77714/328796 |
|  | Essential hypertension | 4.6E-3 | 0.077 (0.027) | 77465/328796 |
|  | Abdominal pain | 4.6E-3 | -0.90 (0.032) | 41128/366074 |
|  | Simple and unspecified goiter | 5.0E-3 | 0.69 (0.24) | 599/389743 |
|  | Hyperplasia of prostate | 6.5E-3 | 0.16 (0.060) | 11176/169004 |
|  | Genital prolapse | 7.4E-3 | 0.15 (0.057) | 11787/207910 |
|  | Cough | 7.5E-3 | -0.30 (0.11) | 2866/398128 |
|  | Frequency of urination and polyuria | 7.5E-3 | 0.25 (0.095) | 4022/383301 |
|  | Pyogenic granuloma | 8.2E-3 | 0.83 (0.32) | 361/395973 |
|  | Uterine leiomyoma | 8.9E-3 | 0.16 (0.061) | 10309/203951 |
|  | Disorders secondary to childbirth, surgery, trauma | 9.4E-3 | -0.90 (0.35) | 302/207908 |

* Association statistics for rs209181 which is in high linkage disequilibrium (r2>0.8) with the top variant at this locus, indel rs146924495, are shown for hematuria

**Supplementary Table 8**. Estimates for hematuria and uACR as predictors in regression models alongside sex, birth year and the first four principal components of genetic ancestry as covariates with the genotype at the genetic variant of interest as the outcome in the UK Biobank. The regressions were carried out an unrelated subset of UK Biobank White British participants.

|  | **Hematuria** | | | **uACR** | | |
| --- | --- | --- | --- | --- | --- | --- |
| **Top variant (associated gene) used as the output in the model** | **Estimate** | **Standard Error** | **P-value** | **Estimate** | **Standard Error** | **P-value** |
| rs35138315 (*COL4A4*) | 1.1E-02 | 5.6E-04 | < 2E-16 | 2.8E-06 | 1.5E-06 | 0.067 |
| rs45551835 (C*UBN*) | 1.6E-02 | 3.9E-03 | 2.5E-05 | 3.6E-05 | 1.1E-05 | 6.6E-04 |
| rs146676616 (*ETV1*) | 1.5E-02 | 3.1E-03 | 1.2E-06 | -1.2E-05 | 8.4E-06 | 0.15 |
| rs146676616 (*TRIM27*) | -6.0E-02 | 1.3E-02 | 3.4E-06 | -2.9E-05 | 3.4E-05 | 0.40 |

**Supplementary Table 9**. Global genetic correlation in the UK Biobank between our composite outcome (estimated heritability: 0.0022 (0.0013)), ICD-based hematuria (phecode 593), uACR as a continuous trait, creatinine-based eGFR (eGFR_creat), and two impedance measurements (whole body fat mass and whole body fat free mass) using summary-statistics-based LD score regression on common HapMap autosomal SNPs and 1000 Genomes European super-population as the linkage disequilibrium reference. The summary statistics for eGFR_creat are the European-ancestry summary statistics from Stanzick et al. 2021. The whole body fat mass and whole body fat-free mass summary statistics are from publicly available UK Biobank summary statistics from the Neale lab.

| Trait 1 | Trait 2 | Phenotypic correlation | Genetic correlation-rg (se); p | Trait 1 estimated heritability on the observed scale (standard deviation) |
| --- | --- | --- | --- | --- |
|  |  |  |  |  |
| Hematuria | uACR | 0.027; p<2.2E-16 | 0.005 (0.0493); p=0.92 | 0.0089 (0.0014) |
|  | Composite | 1; p<2.2E-16 | 0.8495 (0.2471); p=0.0006 |  |
|  | eGFR_creat | -0.035; p<2.2e-16 | 0.0239 (0.0395); p=0.61 |  |
|  | Whole body fat mass | 0.014; p<2.2E-16 | 0.2731 (0.0378); p=5.0E-13 |  |
|  | Whole body fat-free mass | 0.041; p<2.2E-16 | 0.0976 (0.0395); p=0.0134 |  |
| uACR | Composite | 0.34; p<2.2E-16 | 0.6568 (0.1925); p=0.0006 | 0.0528 (0.0026) |
|  | eGFR_creat | -0.025; p<2.2e-16 | 0.3414 (0.026); p=2.1E-39 |  |
|  | Whole body fat mass | 0.0094; p=4.6E-09 | -0.182 (0.021); p= 7.5E-18 |  |
|  | Whole body fat free mass | -0.049; p<2.2E-16 | -0.2014 (0.0228); p=9.1E-19 |  |
| Whole body fat mass | Composite | 0.011; p=1.6E-11 | 0.3072 (0.1238); p=0.0131 | 0.2368 (0.0076) |
|  | eGFR_creat | -0.079; p<2.2E-16 | -0.0639 (0.0158); p=5.2E-5 |  |
|  | Whole body fat-free mass | 0.097; p<2.2E-16 | 0.6304 (0.0127); p=1.0E-308 |  |
| Whole body fat-free mass | Composite | 0.0093; p=5.4E-8 | 0.1036 (0.0819) ; p=0.2057 | 0.2972 (0.012) |
|  | eGFR_creat | -0.0078; p=1.2E-6 | -0.1803 (0.0176); p=1.7E-24 |  |
| eGFR_creat | Composite | -0.035; p<2.2E-16 | 0.3101 (0.1233); p=0.019 | 0.002 (0.0012) |

**Supplementary Table 10. Top signals for composite trait GWAS (HRC-imputed data)** **from UK Biobank analysis excluding individuals taking an angiotensin-converting enzyme inhibitor (ACEi) or an angiotensin receptor blocker (ARB)**
Note that the top variant at the chromosome 2 and chromosome 6 loci remains the same as in the primary analysis in which individuals on medications were included. The *ETV1* and *CUBN* loci no longer pass genome-wide significance, but the direction and magnitude of effects are consistent.

| Chr:pos:ref:alt_b37 (rsID) | Nearest Gene | Effect allele | Primary analysis (n= 2429 cases and 343,509 controls) | | | Excluding individuals on ACEi medications (n= 1658 cases and 298,731 controls) | | |
| --- | --- | --- | --- | --- | --- | --- | --- | --- |
|  |  |  | Beta +/- SE | OR (95% CI) | P-value | Beta +/- SE | OR (95% CI) | P-value |
| 2:227459951_G/A (rs71431010) | *IRS1 ** | A | 16.8 +/- 1.53 | 19286620     (960232-387378891) | 5.97E-28 | 19.2+/- 2.00 | 209210662 (4137490-  1.06E10) | 1.05E-21 |
| 6:28790373_C/CT (rs146924495) | *TRIM27* | CT | -0.22 +/-0.04 | 0.80 (0.74-0.87) | 9.64E-09 | -0.26 +/- 0.05 | 0.77 (0.70-0.84) | 9.87E-09 |
| 7:13349195_G/A (rs146676616) | *ETV1* | A | 0.96 +/- 0.17 | 2.61 (1.87-3.63) | 1.28E-08 | 0.82 +/- 0.20 | 2.27 (1.54-3.34) | 3.05E-05 |
| 10:16932384_G/A (rs45551835) | *CUBN* | A | 0.74 +/- 0.13 | 2.09 (1.61-2.72) | 3.29E-08 | 0.67 +/- 0.16 | 1.96 (1.44-2.67) | 1.81E-05 |

*HRC-imputed UKB doesn’t include the COL4A4 rare LoF variant that is the top signal in the hematuria analysis (assessed in TOPMed-imputed data).  With its implementation of the saddlepoint approximation, SAIGE overestimates the effect sizes of rare variants, but the association p-value remains valid.

**Supplementary Table 11. Top signals for composite trait GWAS (HRC-imputed data)** **with the inclusion of ten principal components (PCs) based on the white British subset in the UK Biobank**Note the significant loci and the top variant at each remains the same as in the primary analysis in which 4 PCs were included. Similarly, the effect sizes and p-values are highly concordant.

| Chr:pos:ref:alt_b37 (rsID) | Nearest Gene | Effect allele | 4 PCs | | | 10 PCs | | |
| --- | --- | --- | --- | --- | --- | --- | --- | --- |
|  |  |  | Beta +/- SE | OR (95% CI) | P-value | Beta +/- SE | OR (95% CI) | P-value |
| 2:227459951_G/A (rs71431010) | *IRS1 ** | A | 16.8 +/- 1.53 | 19286620     (960232-387378891) | 5.97E-28 | 13.3+/- 1.25 | 615095  (53465-  7076309) | 1.07E-26 |
| 6:28790373_C/CT (rs146924495) | *TRIM27* | CT | -0.22 +/-0.04 | 0.80 (0.74-0.87) | 9.64E-09 | -0.22 +/- 0.04 | 0.80 (0.74-0.87) | 8.24E-09 |
| 7:13349195_G/A (rs146676616) | *ETV1* | A | 0.96 +/- 0.17 | 2.61 (1.87-3.63) | 1.28E-08 | 0.94 +/- 0.17 | 2.57 (1.85-3.59) | 2.35E-08 |
| 10:16932384_G/A (rs45551835) | *CUBN* | A | 0.74 +/- 0.13 | 2.09 (1.61-2.72) | 3.29E-08 | 0.74 +/- 0.13 | 2.09 (1.61-2.72) | 2.69E-08 |

*HRC-imputed UK Biobank does not include the *COL4A4* rare LoF variant that is the top signal in the hematuria analysis (assessed in TOPMed-imputed data).  With its implementation of the saddlepoint approximation, SAIGE overestimates the effect sizes of rare variants, but the association p-value remains valid.


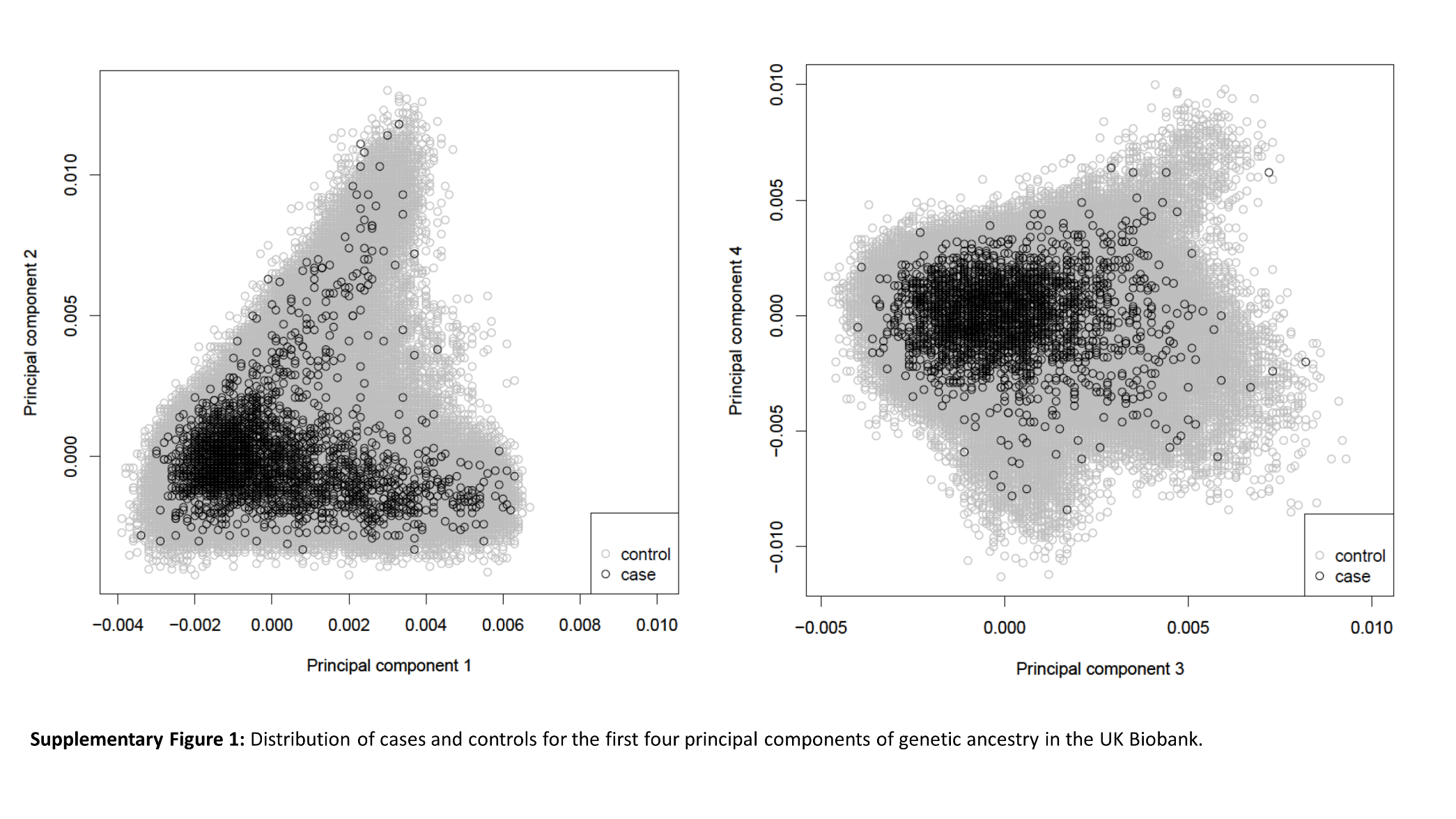


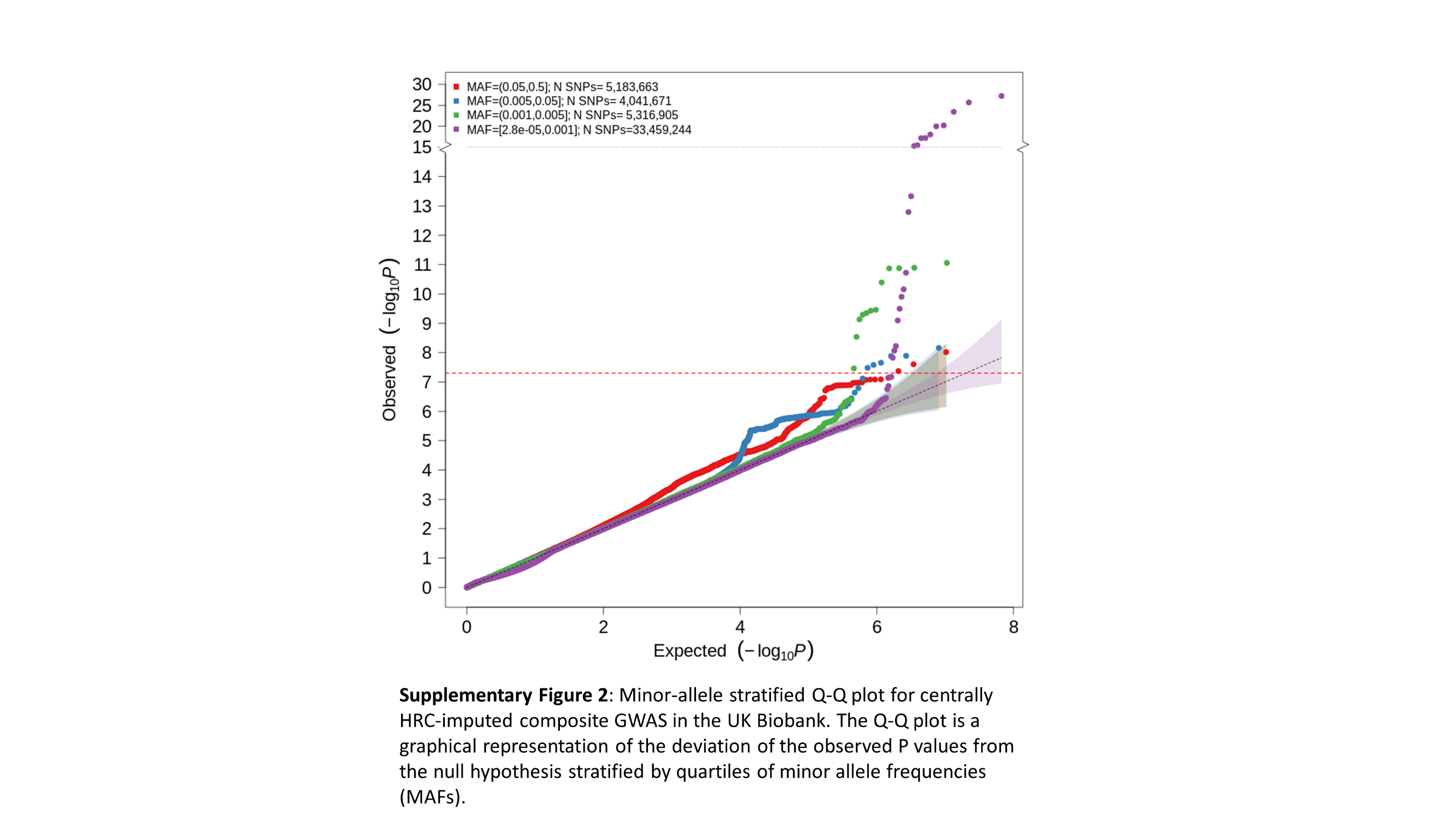


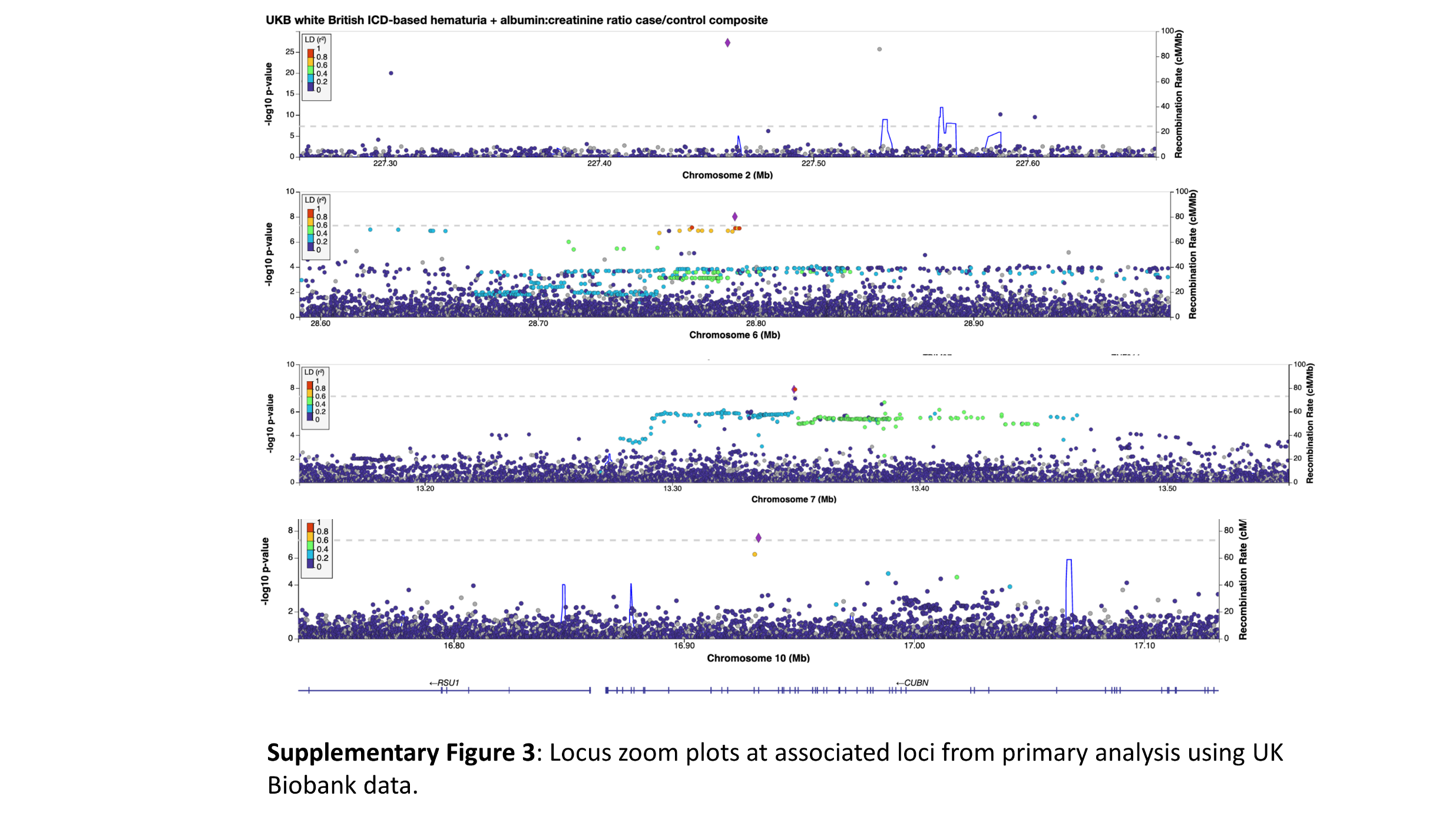


**
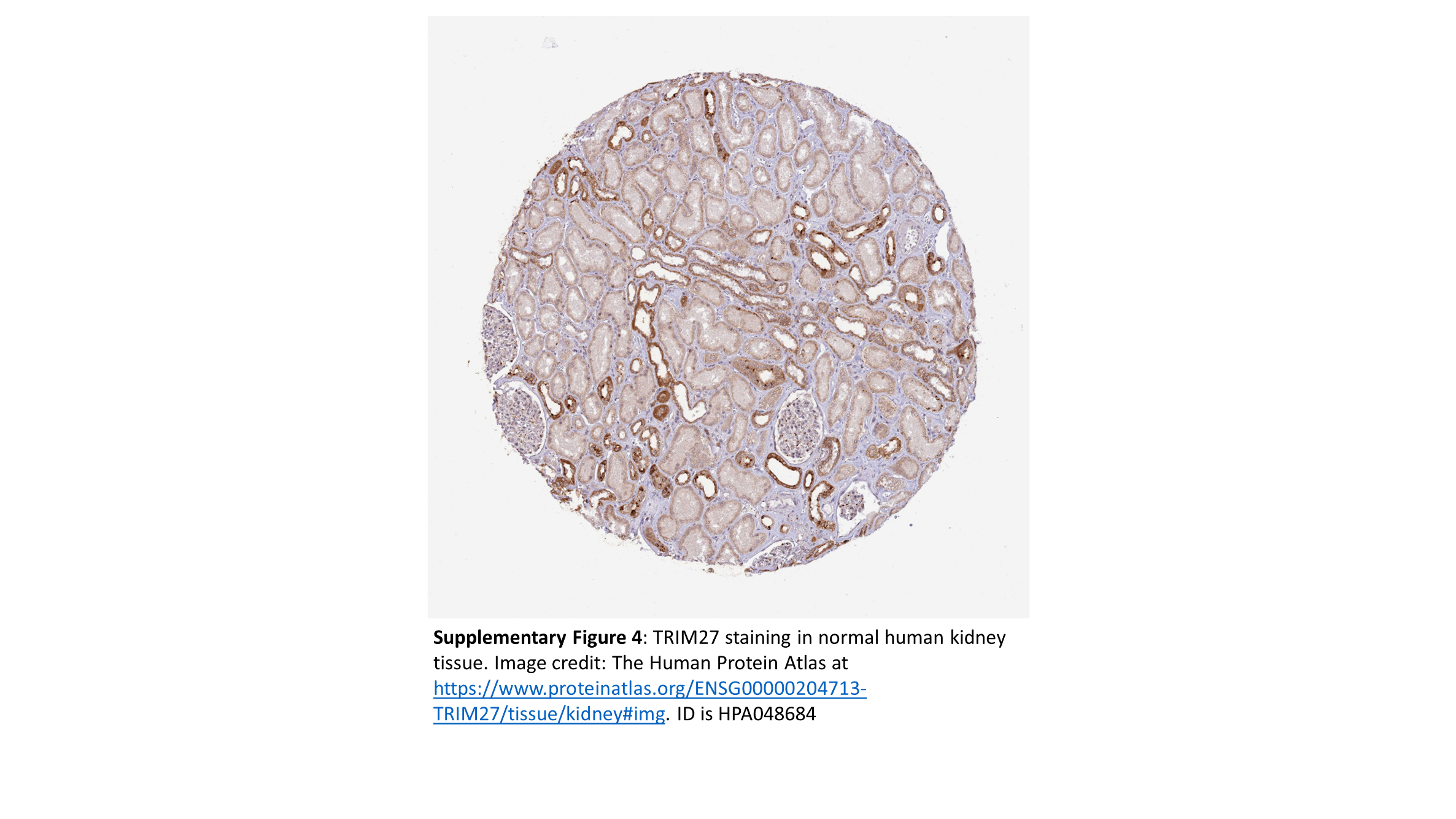
**
